# P-2125. Exploring Risk Factors for Attributable Mortality from Fusariosis in Hematological Cancer Patients

**DOI:** 10.1093/ofid/ofaf695.2289

**Published:** 2026-01-11

**Authors:** David Schantz, Gayathri Krishnan, Anupam Pande

**Affiliations:** Washington University School of Medecine, St. Louis, MO; Washington University School of Medicine, St. Louis, MO; Washington University School of Medicine, St. Louis, MO

## Abstract

**Background:**

While risk factors for acquiring invasive fusariosis in patients with hematological cancers have been studied in the past, the risk factors for attributable mortality are not defined, especially in the United States. This study explored the risk factors for attributable mortality in proven invasive *Fusarium* infections in patients with hematological cancers.

**Table 1:**
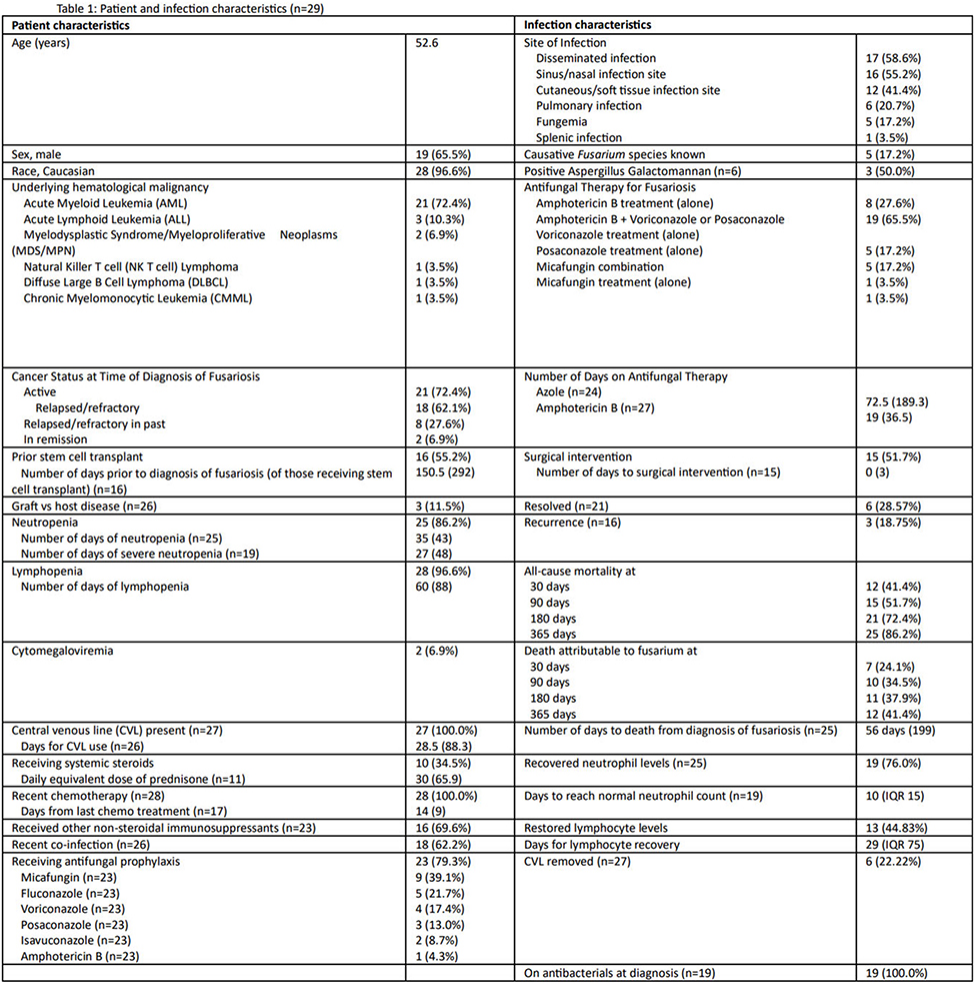
Patient-specific and Fusarium infection-specific characteristics. This table describes the patient- and infection-specific characteristics of a cohort of patients with proven Fusarium infections and hematological malignancies.

**Table 2:**
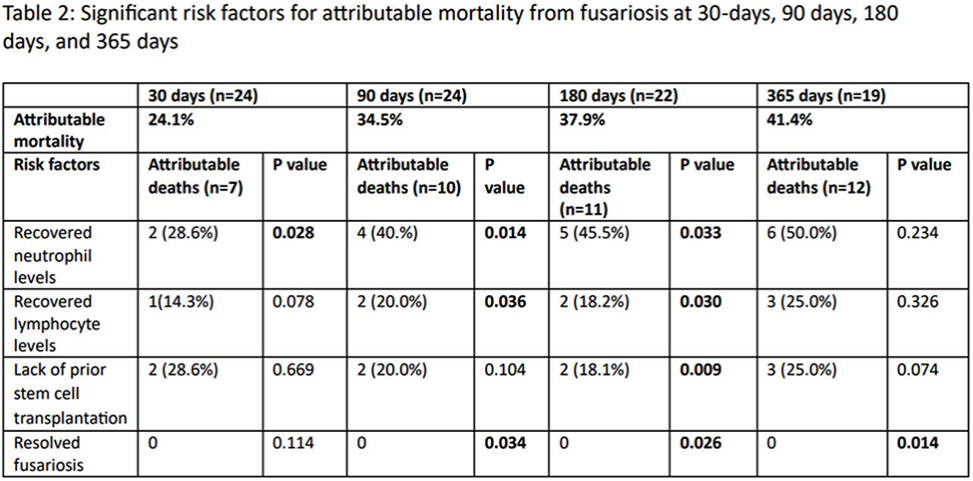
Significant risk factors for attributable mortality from fusariosis at 30-days, 90 days, 180 days, and 365 days This table highlights the significant risk factors for attributable mortality in patients with proven fusariosis and hematological malignancies across various time points such as 30, 90, 180, and 365 days.

**Methods:**

This is a single center retrospective study of adult patients with hematological malignancies and proven invasive *Fusarium* infections admitted to a tertiary academic center from January 2010 to January 2021. Primary endpoint was attributable mortality at 30 days from diagnosis of infection, with secondary endpoints of the same at 90, 180, and 365 days. Statistical analysis was done using Fischer’s exact test and Mann-Whitney U test.

**Results:**

31 patients with hematological cancers and proven *Fusarium* infections were identified: two being excluded due to missing data. Analysis of baseline patient and infection characteristics for fusariosis (TABLE 1) confirmed previously known risk factors such as acute leukemia (82.7%), relapsed/refractory cancer (62.1%), prior stem cell transplantation (55.2%), neutropenia (86.2%), lymphopenia (96.6%), and disseminated infection (58.6%). 30-day attributable mortality was 24.1%. At 30 days, lack of neutrophil recovery was the only significant risk factor for attributable mortality (p=0.028), but at 90 and 180 days, lack of lymphocyte recovery is also significant (p=0.036 and 0.030 respectively) in addition to neutrophil recovery (TABLE 2). Receipt of prior stem cell transplantation was associated with attributable mortality at 180 days (p=0.009) while lack of resolution of *Fusarium* infection was associated with attributable mortality at 90, 180, and 365 days (p=0.038, 0.026, and 0.014 respectively).

**Conclusion:**

This is the largest single center cohort of adult patients with proven *Fusarium* infections in the setting of hematological cancers in the United States studying risk factors for attributable mortality at various time points after infection. Lack of neutrophil recovery was a significant risk factor for attributable mortality in our patients at 30, 90, and 180 days. Lack of lymphocyte recovery was a significant risk factor for attributable mortality at 90 and 180 days.

**Disclosures:**

All Authors: No reported disclosures

